# Plant sterols induce intestinal tumor formation in gender-related manner in ApcMin mice

**DOI:** 10.1186/1753-6561-6-S3-P34

**Published:** 2012-06-01

**Authors:** Maija Marttinen, Anne-Maria Pajari, Essi Päivärinta, Markus Storvik, Mikael Niku, Tanja Nurmi, Vieno Piironen, Marja Mutanen

**Affiliations:** 1 Department of Food and Environmental Sciences, Division of Nutrition, University of Helsinki, Helsinki, Finland; 2 School of Pharmacy, University of Eastern Finland, Kuopio, Finland; 3 Department of Veterinary Biosciences, University of Helsinki, Helsinki, Finland; 4 Department of Food and Environmental Sciences, Division of Food Chemistry, University of Helsinki, Helsinki, Finland

## Background

Plant sterols are plant derived dietary compounds that are structurally similar to cholesterol. Plant sterols reduce cholesterol absorption, and therefore plant sterol enriched functional foods are designed to lower blood cholesterol level. Reduction of cholesterol absorption increases the level of intraluminal cholesterol, and high intraluminal cholesterol concentration has been associated with enhanced cell proliferation, aberrant crypt formation and tumor formation [1,2]. The aim of this study was to investigate, how plant sterols affect intestinal tumorigenesis, sterol composition of the faeces and the intestinal mucosa, and cell signaling in tumor-prone *Apc*Min mice.

## Materials and methods

The *Apc*Min mouse is a well-characterized model for studying associations between dietary factors and colon cancer development. *Apc*Min mice were fed either a high fat, low fiber AIN93-G based control diet (n=12) or a 0.8% (w/w) plant sterol enriched diet (n=12) for 9 weeks. Plant sterol esters were added to the diet in plant sterol enriched margarine. At the end the diet period, intestinal adenomas were counted, and samples were collected for sterol, Western blot, and gene expression analyses.

## Results

The number of intestinal tumors increased significantly in plant sterol fed female *Apc*Min mice (46.8±6.7) compared to control female mice (35.0±9.1). In male, there was no difference in the number of tumors between the plant sterol and control group (41.1±8.2 and 36.3.0±8.5, respectively). No difference in the size of intestinal tumors was observed between the experimental groups. The faecal cholesterol concentration increased by 3.4-fold after plant sterol feeding. The level of mucosal cholesterol decreased in plant sterol fed male compared to control male (-18%, *p*=0.01) and plant sterol fed female (-13%, p=0.028). There was no difference in the mucosal cholesterol level in female between groups. Plant sterol feeding increased the level of plant sterols in the intestinal mucosa, and in male resulted in 2-fold higher mucosal sitosterol level compared to female. No difference between groups was found in levels of nuclear cyclin D1 or β-catenin, however, nuclear β-catenin was increased in plant sterol fed female compared to plant sterol fed male (2.2±0.2 and 1.3±0.4, respectively; *p*=0.011). Enrichment of regulated genes belonging to the terpenoid backbone synthesis (KEGG pathway database) was detected after plant sterol feeding in female. The upregulated genes of the terpenoid backbone synthesis (*Mvk*, *Pmvk*, *Idi1*) transcribe enzymes of the mevalonate pathway, which produces cholesterol and isoprenoids for the cell.

## Conclusions

Plant sterols accelerated intestinal tumor formation in *Apc*Min mice, and the effect was mainly seen in female. In female, plant sterol feeding upregulated gene expression of several enzymes in cholesterol biosynthesis. Our study suggests that in tumor initiation plant sterol enriched diet has no effect in male but is harmful in female mice.
